# Institutional context and VCT practitioner narratives: possibilities and limitations for HIV prevention in Rio de Janeiro, Brazil

**DOI:** 10.1186/s12914-017-0139-x

**Published:** 2017-12-04

**Authors:** Claudia Mora, Simone Monteiro, Carlos Otávio Fiúza Moreira

**Affiliations:** 1 State University of Rio de Janeiro, Rio de Janeiro, Brazil; 2 Oswaldo Cruz Institute – FIOCRUZ, Rio de Janeiro, Brazil; 3 National School of Public Health – FIOCRUZ, Rio de Janeiro, Brazil

**Keywords:** HIV, Counseling, Testing, Health providers, Sociology, Vulnerability, Qualitative study

## Abstract

**Background:**

Voluntary Counseling and Testing (VCT) is an HIV prevention strategy that promotes the principles of confidentiality and informed consent. International research has highlighted VCT counselors’ isolation from service planning and the contradictions they negotiate between local values and global testing recommendations. In Brazil, studies have identified many limitations, including counselors’ difficulties to implement a vulnerability approach to HIV prevention as recommended in the country’s national guidelines. These studies, however, have not considered the particularities of the institutional contexts where counselors work. This research addresses these gaps in the VCT literature by exploring how VCT services are organized and how counselors perceive and perform their practices in the state of Rio de Janeiro, Brazil.

**Methods:**

This is a case study of VCT services in the state of Rio de Janeiro. The research design included individual structured interviews with seven VCT service coordinators and twenty individual semi-structured interviews with VCT counselors. Participants were sampled according to gender, undergraduate degree and work trajectory to capture a diverse range counselor narratives.

**Results:**

The VCT services were relatively homogenous in terms of functioning and had a similar restricted roll of activities including individual counseling and occasional external prevention activities with groups vulnerable to HIV. All VCT services reported reductions in staff size. Some counselors used dialogical practices to build trust, guarantee confidentiality and adjust their practices in accordance with their clients’ values and practices. Others emphasized imperative messages or focused on risk and individual responsibility. Connections between how counselors perceive their practices and the organization of their work environment were observed.

**Conclusions:**

Due to the importance of counseling as a prevention strategy we recommend rethinking the relationship between counselors’ practices and the organization of VCT services. The challenges brought about by the expansion of “test and treat” programs globally and other social and symbolic aspects of the HIV epidemic, such as gender inequalities, must also be taken into account. Further reflection is also needed on the relationship between counseling guidelines and practices within the vulnerability approach to HIV prevention.

## Background

Counseling is a psychosocial intervention with potential for a synergetic individual-community level approach towards social norms and practices [1, 2]. Its use in the context of HIV prevention strategies and diagnosis, referred to as Voluntary Counseling and Testing (VCT), is based on vulnerability and human rights frameworks. In fact, the vulnerability approach revealed the importance of social determinants and structural factors in spreading the AIDS epidemic [3]. Based on the principles of confidentiality and informed consent VCT strategies have been implemented in multiple countries over the past three decades, including Brazil, Argentina, the United States, Malawi, Uganda, Egypt, China, and Switzerland.

The United Nations General Assembly Special Session on HIV/AIDS (UNGASS) committed to expanding access to anti-retroviral therapy (ART) in 2001 [4]. The UNGASS position motivated the WHO and UNAIDS to implement the “*scaling up testing”* initiative throughout the decade of 2000. The expansion of testing sparked an international debate synthesized through two distinct positions. The first position refers to the preservation of the *paradigm of exceptionalism* in the test to detect HIV. This paradigm is based on the principle of autonomy and human rights and seeks to promote prevention actions through counseling and testing. The second position refers to the *normalization of testing* and values the collective benefits derived from the access to treatment to the detriment of certain rights like individual autonomy [5].

More recently, Treatment as Prevention (TasP) recommendations from the WHO and UNAIDS [6, 7] have contributed to expanding testing and discussions regarding the need to make the principles underlying VCT more flexible. This debate resulted in proposals to diversify testing with an emphasis on increasing access among populations most vulnerable to HIV (ex. sex workers, men who have sex with men and drug users). Such proposals generated several technical and ethical challenges including how to guarantee confidentiality and informed consent [8], and how to avoid reinforcing unequal power relations already present within clinical practices [9].

The literature on STD/HIV counseling and testing service practitioners concentrates on health professionals’ experiences, giving less attention to their practices. Such research highlights the difficulties professionals face, such as overworking or frustration, as a result of a lack of resources (material and human), physical space and long hours. In addition, some studies point to limitations in VCT service planning and decision making due to the lack of recognition of counselors by local health authorities [10–14].

Other studies discuss how global prevention recommendations conflict with the sociocultural contexts where VCT services operate [15, 16]. Angotti’s [17] research in Malawi illustrates how professionals balanced the principles of individual autonomy to be tested with community values of well-being by promoting collective, as opposed to individual, testing campaigns.

Brazil adopted the VCT strategy in 1988 and since then, VCT services have become a reference for HIV diagnosis and treatment within the country’s Universal Health Care System (SUS – acronym in Portuguese). As of 2010, there were 515 VCT services throughout Brazil offering prevention and testing activities, especially for the groups most vulnerable to HIV (sex workers, men who have sex with men and drug users) [18]. Recent data indicates that 38% of AIDS diagnoses are late-stage, and 52.1% of all cases identified between 2007 and 2016 (71,396) are concentrated in the Southeast region, which includes the state of Rio de Janeiro [7].

Convergent with the “*scaling up testing”* initiative and the expressive percentage of late stage diagnoses, testing strategies have come to form a central part of Brazil’s response to HIV/AIDS. As part of the country’s efforts to decentralize HIV testing over the past decade, rapid tests have been made available in a variety of spaces, including primary health care services, maternity wards, urgent care clinics, and more recently non-governmental organizations.

Analyses of Brazilian VCT networks have found some benefits in terms of access to testing and dissemination of information about STD/AIDS [19]. Various studies, however, have also identified several limitations in terms of incorporating an intersubjective perspective and vulnerability approach [3, 20] to HIV testing and counseling as stipulated by the Brazilian Ministry of Health [21–23]. Pupo and Ayres [24], for example, point out two implicit competing rationalities in the Ministry of Health’s counseling recommendations [25]. On the one hand, the recommendations reinforce counseling theory based on a Person Centered Approach, that brings together the notion of counseling from the Humanist School and support actions developed by social movements in the 1970s (such as the feminist and gay movement) [25], and prioritizes promoting constructive changes in the clients’ lives. On the other hand, the guidelines promote public health goals centered upon universal prevention assumptions such as using condoms in all sexual relationships and notifying partners.

This study explores how VCT services are organized and how counselors perceive and perform their practices in the state of Rio de Janeiro. Based on interviews with seven VCT center coordinators and twenty counselors, we analyze two key aspects of counselors’ practices: the institutional context of the VCT centers included in the study (infrastructure, organization and activities, team, days and hours) and the counselors’ narratives of counseling and prevention.

The study draws on Bourdieu’s [26] concepts of habitus and the field. Understood as *know how* (a form of practical knowledge), habitus results from socialization processes and is expressed through social agents’ ways of doing, perceiving, classifying, and feeling. A field refers to a part of society that has its own rules, logics of action and own means of reproduction, such as training processes in the fields of Education, Law, and Health. The relationship between these two constructs is mutually constitutive as both parts are of a social nature. In short, habitus refers to the intersubjective dimensions of action and the field informs its structural aspects. Our research is centered upon a hypothesis that the counselors’ *professional habitus* expresses a relationship between the objective and intersubjective dimensions of their practice, which in turn structures how they approach clients during counseling. In this sense, we seek to interpret the relationship between their narratives of practices and the contextual characteristics of the services where they work [27].

## Methods

### Design and participants

The research presented here is a case study [28] of VCT services in the state of Rio de Janeiro. The study design includes individual structured interviews with VCT service coordinators and individual semi-structured interviews with counselors. VCT services were sampled based on geographical characteristics in that the study contemplates all of the Rio de Janeiro health department sub-regions with VCT services. Of the nine sub-regions, five have VCT services, of which three sub-regions had one service and two had between two and three services. In the regions that had only one service, that service was included. In the regions that had more than one service, the oldest services (implemented in the 1990s) and the most recent services (implemented in the 2000s) were prioritized.

As part of the study, first seven VCT service coordinators participated in an individual structured interview regarding certain aspects of service organization including hours, supplies, available exams, client profile, staff organization and training. Data from the interviews were registered in a field diary.

The information provided by the coordinators regarding the composition of their teams was systematized according to the staff’s schooling, sex and time working at the center. The profile of the VCT teams was primarily female, with educational backgrounds in health and the humanities and between one and twenty years working in the VCT services. We purposefully sampled counselors to be interviewed to ensure a sample as representative as possible of the VCT service teams considering their training, time in the VCT service and sex. In each service we interviewed at least one professional with a background in health, one from the humanities in addition to one with more experience and one with less experience. As a large part of the counselors were female, we recruited male counselors in an effort to capture narratives from both sexes.

The first author conducted a total of twenty semi-structured interviews with counselors between October 2011 and July 2012. The interviews were conducted at the VCT services and focused on their professional practices and viewpoints in relation to prevention, sexuality and AIDS. Each interview was conducted in a single session lasting approximately 60 min and participants all signed an informed consent form. The interviews were audio recorded, transcribed and the transcripts were organized with the assistance of MAXQda software.

In general the interviews had few interruptions due to the low number of clients. When describing their practices, counselors shared and described routine materials they used, such as forms and educational pamphlets. Discomfort related to service management was discussed when the recorder was turned off.

The counselors’ age bracket varied between thirty and sixty years old and included seventeen women and three men. Most counselors work twenty hours per week in the health center’s municipality. The health providers’ undergraduate degrees were concentrated in Nursing/Nursing Technician (10), followed by Psychology (4), Social Work (3), Physiotherapy (1), Pharmacy (1) and Sociology (1). Most interviewed were involved in STD care and prevention due to their employment relationship with the VCT, which spanned between one and twenty years (Table 1).

**Table 1:** Socio-demographic profile of counselors interviewed

Pseudonyms	Sex	Undergraduate degree	VCT work trajectory (years)
Aline	F	Nursing technician	11
Bianca	F	Physical therapist	5
Camila	F	Pharmacy	7
Danielle	F	Social service	19
Edna	F	Social service	1
Francisca	F	Nursing Technician	0.5
Gabriela	F	Sociology	20
Helga	F	Nursing	4
Irma	F	Psychology	19
Joana	F	Psychology	8
Kevin	M	Social Service	7
Luana	F	Psychology	6
María	F	Nursing	3
Nino	M	Nursing	5
Olga	F	Nursing technician	10
Paola	F	Nursing	1.5
Quésia	F	Psychology	0.5
Roberto	M	Nursing	18
Sandra	F	Nursing	5
Tatiana	F	Nursing technician	5

### Data analysis

Data was analyzed using thematic analysis [29]. The first step consisted in systematizing socio-demographic and organizational characteristics. At the same time, the material was read repeatedly to create familiarity with the content. Then, narratives were classified into thematic categories by the first and second authors. These categories included counseling notions and experiences, client-counselor interactions, prevention activities and perceptions about AIDS and sexuality. To control bias, the first and second authors wrote descriptions of the thematic categories and then a third author discussed those descriptions to resolve meaning discrepancies and support data consistency. The first author then coded the transcripts, organized them in accordance with the empirical categories and analyzed the corpus of the results.

Our findings are organized in two dimensions; the institutional context of the services studied and the professionals’ perceptions of STD/AIDS counseling in light of their institutional contexts. We start with the institutional context because we consider it as a factor that structures the counselors’ practices.

## Results

### Institutional context of the VCT services

In this section we present the characteristics of the VCT services included in the study. For more details of each service researched, including a nomination letter to each one (A, B, C, D, E, F, G), see Table 2 at the end of this paper.

**Table 2:** Characteristics of the seven VCT researched

Sub-region-VCT	Year of implementation	Team	Internal activities (with vulnerable population groups, general population, harm reduction)	External activities (with vulnerable population groups, general population, harm reduction)	Prevention supplies	Tecnological profile^a^
Sub-region I						
A	1992	4 counselors 1 receptionist	Individual counseling Rapid test: HIV, syphilis, HVB, HVC. Client-initiated Testing center for pregnant women and clients with tuberculosis	Not reported	Male condoms	Diagnosis
B	1995	1 counselor and 1 support counselor 1 nursing technician	Individual and group counseling Rapid test: HIV, syphilis, HVB, HVC. Client-initiated Testing center for pregnant women and TB	Not reported	Male condoms	Diagnosis
Sub-region II						
C	1992	5 counselors 2 receptionists	Individual and group counseling Conventional test: HIV, syphilis, HVB, HVC HIV rapid test Client-initiated	Intervention with sex workers	Male and female condoms	Care and Prevention
D	1995	3 counselors 4 receptionists	Individual counseling Conventional test: HIV. Occasional offer tests for syphilis, HVB and HVC Client-initiated	School interventions	Male and female condom	Diagnosis
Sub-region III						
E	1993	1 counselor and 1 more for support	Individual counseling Conventional test: HIV, syphilis, HVB, HVC Client-initiated	Intervention with harm reduction program for drug users	Male and female condom	Diagnosis
Sub-region IV						
F	No date	12 counselors 4 receptionists	Individual and group counseling Conventional and rapid test: HIV, syphilis, HVB, HVC Client-initiated	Mobile VCT service		Care and Prevention
Sub-region V						
H	1992	2 counselors 1 receptionist	Individual counseling Conventional and rapid test: HIV, syphilis, HVB, HVC Client-initiated	Educational actions with regional businesses and schools	Male condoms	Diagnosis

^a^According to the Brazilian VCT diagnostic study (Brazilian Ministry of Health 2008) each service could have four distinct technological profiles (Prevention, Care and prevention, Diagnosis, and Care)

We compare the results with various indicators from the Brazilian Ministry of Health’s 2008 diagnostic study of VCT networks [30]. These indictors include year established, populations reached, internal and external activities, prevention supplies distributed, professional team characteristics, hours and prerequisites to be seen at the service. Through this comparison, we seek to characterize the type of VCT service in which the counselors work according to the profiles established in the 2008 report of the Ministry of Health’s diagnostic study: *Prevention, Care and prevention, Diagnosis,* and *Care*. In general, these profiles can be distinguished from one another by the capacity to offer counseling and testing for vulnerable population groups or the general population. They also differ through the realization of diverse activities such as individual counseling, harm reduction, and STD treatment, among others. Although there is not a “gold standard profile” established in the Ministry of Health’s report, identifying the specific benefits and limitations of each profile was considered strategic for thinking about how to expand testing coverage in Brazil. We detail the characteristics of each VCT service in the next section.

The Ministry of Health established VCT services as alternatives to the standard public health network. We found, however, that they were organized in a similar fashion to conventional health centers. For example, according to the seven VCT service coordinators, they operate with only day-time hours, during the work week, and consultations were regulated through the distribution of numbers at the beginning of each shift or prior scheduling. Most services were located within other health centers and only had rooms for individual consultations. Audiovisual resources were scarce and few VCT services had telephones or internet to facilitate contact with the public. The male condom supply was continuous, but other prevention supplies, such as lubricant and female condoms, were not available.

The national norms that regulate VCT services stipulate conducting actions within and outside the structure of health services and offering different modalities of counseling (individual, group, couples, seropositive and sero-discordant). The norms also recommend the availability of counseling for people awaiting exam results (HIV, syphilis, hepatitis C, hepatitis B) and for people and families living with HIV/AIDS and viral hepatitis [18].

Although the predominant form of HIV transmission in Rio de Janeiro was sexual, affecting mainly men who have sex with men (MSM) and young women, [31], the offer of services for these populations was nearly non-existent. Women were only prioritized for VCT services when pregnant and only one VCT service (F) offered night services and conducted activities outside the clinic. This situation facilitated access and health promotion activities for sex workers and the transgender population.

In general, contact with the VCT services is client-initiated in accordance with the WHO’s Client-Initiated HIV Testing and Counseling (CITC) approach [32]. In other words, service use depends on a person’s deliberate and voluntary decision to seek it out (referred to in Table 2 as “client-initiated”). To some extent, all of the services also received clients referred by practitioners from other health services. Two VCT services (A and B) offered specific time periods exclusively for pregnant women and people with tuberculosis. This variation in functioning deviates from the established mission of VCT services: to facilitate access to information or active listening without needing to schedule an appointment or a pre-existing condition [18].

Individual counseling predominated the services offered. Although Brazil’s national guidelines recommend forming treatment adherence groups, none of the VCT services reported having them at the time of the study. According to the VCT service coordinators, limited staff makes difficult to conduct all of the recommended actions. Some counselors interviewed mentioned that this activity was conducted only when there were more professionals on staff (Ministry guidelines stipulate having at least four professionals on staff for these kinds of activities [18]. As one of the participants commented (hereafter counselors’ narratives will be identified with a pseudonym and their professional training, see Table 1):

It became difficult to do group therapy, individual therapy. They (seropositive clients) had to wait until all of the testing services ended to be seen, and often they work. (Irma, psychologist)

Three of the seven services (B, C, F) offered group counseling; this activity is defined as “a participatory educational process that seeks to awaken/expand the client’s risk perception to identify the signs and symptoms of STD/HIV/AIDS” [25]. In service F this practice was a requirement for testing. In centers C and B, it depended on the professional on call’s opinion of the practice and the presence of at least three or four clients in the waiting room. The counselors, however, believed that the informative approach of group counseling discourages the public for different reasons. For example, one counselor shared how after explaining the modes of transmission and immunological window some clients decide not to be tested:

While we were explaining what HIV was, how its transmitted, etc., he stopped to think that he had been exposed to a risk a week earlier. So then he said, “it’s not time for me to be tested”. And another client said: “I’m not prepared to receive a positive result today”, and he also stood up and left. (Tatiana, nursing technician)

As a counterpoint, four counselors with degrees in the humanities shared their efforts to use participatory approaches as a way to stimulate a group conversation about STDs and gender stereotypes:

I don’t let people leave without prevention work, because it’s more fundamental than giving a positive or negative result: you have to put something in their consciousness, like television does. I have experience for that, I earn that for that, I do the best I can. (Gabriela, sociologist)

In terms of cross-sector partnerships, those interviewed discussed specific actions implemented alongside programs connected to the mental health and educational sectors. Yet, they did not mention any consolidated partnerships. Such gaps compromise the VCT services’ ability to conduct activities outside the services and limit their recognition as prevention services integrated into the AIDS response in the mid and long term.

Despite the seven services being relatively homogenous in terms of functioning, five of them had characteristics of *Diagnosis* profile [19]. That is, these VCT centers (A, B, D, E, G) offered individual counseling, operated during daytime hours, primarily attended to pregnant women and clients referred from clinicians, offered HIV diagnoses and may have also offered syphilis and viral hepatitis diagnoses. These services are deficient in establishing referrals for care and have restrictive criteria for access, such as mandatory identification and pre-test counseling. There was also little space to discuss planning and service functioning, which helps to understand why Ministry of Health guidelines [18] had not been incorporated in their workflow.

Only two of the VCT services (C, F) came close to the *Prevention and Care* profile meaning that they conducted actions beyond testing, such as: alternative hours, care and prevention actions for vulnerable population groups and extended offering of STD treatments. In these services, counselors had a reasonable level of participation in the planning and realization of internal and external activities, including the implementation of the Ministry’s guidelines [18].

In our contextual analysis of VCT centers, we identified individualized approaches to counseling and limited schedule flexibility as organizational aspects that constrained the type of practices conducted in the VCT services.

### Perceptions of counseling

In our analysis we identified elements that practitioners highlighted as important regarding their interactions with clients and their viewpoints of prevention. We also observed the relationship between the organization of the VCT services and the consultation dynamics.

#### Dynamics and interactions between counselors-clients

Pre-test counseling was reported to begin with the counselor introducing themselves and the service to the client. After that, counselors fill out the form for the VCT information system (SI-CTA in Portuguese)^,^ which includes risk behavior and STD exposure information. Each counselor mentioned using the form in a different way, be it in terms of the order they ask the questions or how the addressed certain topics. For example, some counselors said they asked more direct questions while others sought indirect ways to address intimate topics such as the number of sexual partners. According to the majority of interviewees, pre-test sessions tended to last between ten and thirty minutes.

For most counselors, the VCT information form is relevant because it legitimates broaching intimate topics and registers valuable information for subsequent orientations. Yet, they also mentioned that filling out the form impinged a script to their interaction with clients and occasional differences between the information heard and the information documented on the form. The reliance on the form as a script for counselor-client interactions was seen as limiting the extent to which counselors could explore more subjective questions with clients, and possibly compromising the goals of the public health service.

Counselors shared that each professional has their own style in terms of more or less formality in their approach, the use of the VCT information form and how they develop dialogue with clients:

The biologist is very quick in the interview. When the psychologist was attending, she was also very objective. Not that they didn’t try, they sought [information] in another way. I try to make it so that the person perceives that they can feel good to talk here. Sometimes I succeed, sometimes I don’t. (Luana, psychologist)

I think that artists have to go where the people are and that you have to talk so that the people here [VCT service] understand something. I tell jokes to see if I can bring them to me (Bianca, physical therapist)

Independent of the veracity attributed to the client’s explanation, the counselors reported emphasizing the principles of confidentiality and privacy in their face to face interactions. In the words of one of the participants:

I have to say that the interview will result in a statistic, but that this information is not going to leave here [the VCT service]…because they ask (Tatiana, nurse technician)

This principle is related to the *modus operandi* of some services that display posters reminding the clients: “Everything you saw or heard here should be left here”. The importance of trust and confidentiality also contributed to identifying other demands, such as situations of gender violence or restarting ART. According to the interviewees, attention to such situations does not occur in other testing units, such as private laboratories or other standard health services, where counseling is not offered. Counselors defined clients’ test seeking behavior as an act of *“*courage” due to stigma and prejudice associated with AIDS and identities or practices considered as deviant. Some counselors also identified resistance or reservations among clients to discuss intimate issues and said they use communication strategies (ex. “jokes”, “ice breakers”) to establish trust.

According to some, clients have experiences that lead them to believe they’ve contracted the virus. Counselors were aware that clients see them as “strangers” and highlighted the importance of creating a favorable environment to allow the client to “say what they have to say”. Some interviewed further emphasized the singularity of care offered, comparing it to a doctor’s approach:

The way of we welcome patients…is very different from the doctor. ‘Good morning! Is this your first time here? [Did you come] because you want to or someone asked you to?’ Sometimes it can be because a new partner asked them to come. People need attention (Luana, psychologist)

In addition to earning trust in pre-test counseling, counselors also recognized the importance of discussing clients’ trajectories more broadly and reconstructing the situations that lead them to be tested. They mentioned that more personalized attention was limited by the services’ hours, number of practitioners and lack of computerized VCT information system.

Counselors reported not having a script to guide post-test counseling and that exam results were either given directly or after preliminary considerations regarding the client’s testing motives. They also shared that counseling for negative results tends to be brief (between five and fifteen minutes). They felt that clients’ strong emotions at receiving their results often compromised any reflections they might reinforce from the pre-test counseling:

Any type of emotion is very intense and they don’t pay attention at all… [when it’s negative] they are very happy, very satisfied and thankful and leave (Kátia, nurse technician)

On the other hand, when the test result is positive, counseling was reported as tending to be longer, approximately one hour. Professionals mentioned supporting the client in assimilating the result and encouraging their adherence to ART.

We found the professionals’ undergraduate degrees influenced their counseling practices. Those trained in psychology (4) took intersubjective aspects of counseling, such as the client’s situation, into account and emphasized the importance of clients expressing their feelings when they receive the result. They reported empathetic behavior and physical contact to comfort clients, such as hugging, holding their hands and crying together. For this group, emotional support seemed to represent an end in and of itself.

For professionals trained in nursing (6), establishing emotional stability was important for the clients to assimilate the medical indications. In this sense, one informant, whose service offered the rapid test affirmed that:

Within the questions [from the form] we try and find a way to lighten the mood, to relax the patient and if the result is positive, I need him to be relaxed so that he can understand what we’re going to explain. So we really need to be perceptive, to diminish or even soften [the result] so that he doesn’t go back to being anxious. Sometimes they cry and we need to let them cry, and then do the counseling and referral. (Maria, nurse)

#### Viewpoints on prevention

In this section we focus on individual counseling and present the counselors’ underlying assumptions regarding HIV prevention and their counseling practices. We detail the strategies they used to stimulate condom use and illustrate the forms through which they adjust their practices according to the clients’ values and beliefs.

Authoritative or paternal attitudes emerged in counselors’ narratives describing their prevention discourses in counseling sessions. The professionals often positioned themselves as decision-making advisors, and were confident regarding the efficacy of their own discourses. As a possible effort to intensify their power of conviction, they reported adopting an authoritative tone or emphasizing formal or scientific language.

We found that failure to use condoms revealed in pre-test counseling turned into a justification for counselors to employ an authoritative discourse in post-test counseling. The recommendation to use condoms in the post-test counseling appeared as a prescription:

If they have a risk behavior, it has to change. It would be condom use. People are still very resistant. In our case, we can’t say, ‘go home, lay down and sleep because you don’t have anything’. I prefer to say that a month from now and with all of the recommendations: ‘from now on, you are always going to use a condom!’ The majority of people accept it. (Roberto, nurse)

Other narratives equated condom use with something to be incorporated into daily life along with other hygienic (taking a bath), sensory (trying new food), and esthetic (walking in high heels) care habits. Efforts to re-signify the negative connotations surrounding condom use and incentivize its use motivated demonstrations and metaphors:

I say: ‘Pills are to pregnancy what condoms are to disease.’ (Helga, nurse)

They [clients] tend to say: ‘Ah, but then I lose all my sensitivity’. I go the fridge, get ice, put a rubber glove on them and ask: ‘are you feeling it?’ The glove is even thicker than a condom. I try and convince them this way. (Irma, psychologist)

During counseling I repeat a situation a patient told me: he went to a brothel and realized he had to use condoms; she [sex worker] put it on with her mouth, because prostitutes have this type of relationship with condoms. Did he lose sensitivity? No! (Joana, _Psychologist_)

Some professionals drew attention to how beliefs and values influence the client’s adoption of prevention practices. According to one counselor, for clients who associate religion with protection, it isn’t effective to recommend condom use in all circumstances. The following counselor considered it more important to stimulate reflections about the decision to use condoms and search for other ways of care consistent with her client’s values.

There are people who are Evangelicals, Catholics, but before entering into a religion they had an active sex life. So you have to think about the previous context. And women sometimes think they are immune for being in [a specific] religion, but sometimes their partner isn’t from the same religion. So it is a risk context …Rich, poor, black, white, any religion, the virus in in society and at any moment people can get involved and be at risk. The religious question isn’t about immunity, but it has to be respected, I’m not going to keep saying, “use condoms, you have to use them, etc.”. It has to be the couple’s reflection. If she decides not to use them, or he decides not to use them, they at least need to know the serological status of their partner or why they’re going to use them. (Aline, nurse technician)

In fact, counselors asserted that test seeking was associated with strengthening or breaking affective ties, especially among heterosexual couples. In these cases test seeking might result in abandoning condoms or deciding to use them for specific situations (pre-nuptial, serodiscordant, etc.).

Among couples that separated and got back together one of the two demanded the exam as a condition to get back together. Another client said that having a negative result would be a plus for his life, as he would be able to have a “normal” relationship, because he thought he could dispense condoms. (Camila, pharmacist)

The counselors mentioned dealing with situations of poverty and gender violence. They incentivized clients to make individual decisions to avoid STDs and offered support and/or referred the clients to other health or social services. We observed that professionals trained in social work and professionals who worked in the two VCT services with the *Care and Prevention* profile were more likely to have this type of cross-sector action:

I had a client with a black eye for example, I did her whole consultation, but I didn’t avoid touching on this: shoot, did you fall? Hurt your eye? ‘No, this was my partner, he hit me.’ This makes it possible for me to take another approach with her about violence…we know that the tendency is for the violence to diminish when the abuser is reported and that she should report it to competent agencies to get help through special programs (Kevin, social worker).

The professionals placed strong emphasis on HIV prevention, but in the daily life of the VCT services, they also reported being confronted by situations of imminent positive diagnoses. According to one of the VCT service coordinators, thirty-five per cent (35%) of their clients sought out testing due to presence of symptoms associated with AIDS. Moreover, counselors indicated that clients requested the exam with the expectation of receiving treatment right after diagnosis. Our study was conducted two years before Brazil adopted treatment as prevention [33], so this expectation was not directly influenced by it, but more likely by representations of AIDS as a chronic disease and clients’ understandings of Brazil’s universal access to ART policy. A counselor who reported attending serodiscordant couples stated:

I’ve had people who tell me: ‘I choose not to use condoms; I think that it’s easier to treat HIV than to use them’. I think that the citizen, the client, has the right to choose, but we try to make it so that this choice is the best one, the choice for prevention (Gabriela, sociologist)

With regard to perceptions of safety or protection, professionals stated that clients justify periodic tests (monthly, half-yearly or annual) as a form of “prevention”:

There are those that do it [the test] over and over and over, then screw around and screw around and are always here (Bianca, physical therapist)

We have a few characters that are marked, that we even know, who come here almost every month to be tested. Some of them are at risk, they open one immunological window and close another. (Nino, nurse)

Seven participants understood this situation to be problematic. Considering the temporary relief the clients felt when they received their negative test results, professionals felt that clients started to see the exam as a “vaccine” or a “forgiveness” ritual. They also discussed how serological status represents a form of secondary prevention for some clients. Post-test counseling with a negative result allowed counselors to reinforce primary prevention:

I said to the client: “everything that you were thinking didn’t happen, but from here on out, the responsibility continues to be yours.” Because some people live this: “ah, I’m going to save this (result)”. Yes, you can save it, but it isn’t necessarily a guarantee of anything, it is only a test of how you’re life’s been up until now. The other issue is that there are people who come here every year to do “prevention”, so this isn’t prevention…prevention is what you do. If you know that you’re being careful there is no reason to worry, you don’t need to keep religiously doing the exam every 6 and 6 months. (Quésia, psychologist)

## Discussion

International research has highlighted counselors’ isolation from service planning and the contradictions they negotiate between local values and global testing recommendations. Yet studies have looked less at how such structural barriers affect counselors’ day-to-day work. In Brazil, diagnostic studies have identified many limitations, including counselors’ difficulties to implement a vulnerability approach to HIV prevention. Under this approach, prevention practices consider socio-structural (institutions, material life conditions) and socio-symbolic (gender norms, social expectations) aspects of communities with the goal of promoting significant and long-lasting changes [3, 20].

These studies, however, have not considered the particularities of the institutional contexts where counselors work. This study addresses these gaps in the VCT literature through its exploration of the relationship between institutional contexts and VCT counselor practices.

We identified two concurrent perspectives that crosscut counselors’ practices. One perspective is focused on risk and individual responsibility and the other takes an intersubjective and dialogic approach. We found that the first perspective was reinforced by the VCT service institutional context; services were organized in a way that privileged individual activities and bureaucratic means, such as the VCT form, to script professional activities. Individual counseling was prioritized, and there were few collective activities or efforts to reach vulnerable population groups such as MSM and young women. We also noted a disconnection between clients’ and counselors’ viewpoints of prevention. The second perspective manifested through the counselors’ dialogical practices that addressed the importance of trust and confidentiality. We found that counselors adhering to this perspective adjusted their practices in accordance with their clients’ values, practices and vulnerability context (such as being in stable relationships or their religious beliefs).

Our findings suggest a transformation in the Rio de Janeiro state VCT network. Over the past two decades, it has shifted from prevention actions with material and human resources to a more basic care based model focused on providing diagnoses. Grangeiro et al. [19] came to a similar conclusion in their diagnostic study conducted in the first decade of 2000 and more recent studies have also noted institutional constraints on STD/AIDS counselors’ abilities to provide more personalized and far-reaching support [10–12, 34].

Our findings also suggest a connection between the decentralization of HIV testing to primary care services in Brazil and staff reductions and less attention to the groups most vulnerable to HIV. For example, group counseling was offered in only three of the services studied; a situation similar to other VCTs in Brazil (3.8% of 515 services) [30]. VCT coordinators may have difficulties in integrating their services within the new HIV testing and assistance organization, between primary care, hospitals and specialized care. For clients, such changes might result in new therapeutic itineraries and modified relationships with practitioners. Such changes are concomitant with the prominent expansion of testing at the international level in detriment of prevention actions with a more social and structural basis [35]. More research is needed, however, looking more specifically at how the decentralization of testing has affected Brazil’s Universal Health Care System.

Studies conducted among young people, sex workers and other social groups have highlighted the relevance of community mobilization strategies that include testing and counseling. These approaches differ from the biomedical (ex. post-exposition prophylaxis, circumcision) and behavioral (ex. condom use, harm reduction) models because they combine education, social welfare and empowerment components in a framework in which HIV prevention is directly related to broader citizenship rights [36–38]. Our findings also suggest, however, that VCT services in their current contexts might not have the structures necessary handle a higher influx of vulnerable population groups. This is an additional need to address to guarantee the eventual effectiveness and sustainability of a community mobilization approach.

Regarding the client-counselor interactions, up to a certain point, counselors reporting following the VCT guidelines, but they also adjusted their behavior in accordance with their prevention beliefs and client demands. Our results also demonstrate how counselors relativize clients’ representations of safety (also denominated as “the familiar protects” [39]); some of them interpret clients’ feelings of safety associated with their erotic-affective relationships held as stable.

Counselors interviewed considered the VCT intake form to be an adequate guide for client-counselor interaction. Yet it’s important to recognize that the form was created based on a logic of identifying risk factors [22, 23]. The use of the form in the VCT session stimulates a search for individual risk profiles by bringing together what Biehl et al. [40] refer to as “risk sciences, pastoral dictates and screening technologies”. Reproducing this dynamic without reflection limits understanding clients’ vulnerabilities and therefore the stated purpose of STI/HIV counseling to “help the client to find a viable protection plan” [25]. As we’ve argued elsewhere, VCT counselors’ chances for renovating the *professional habitus* that organizes and reproduces their daily practices are reduced due to a lack of opportunities to reflect on their practices, knowledges and beliefs [13].

Looking forward, we see several findings as important areas for future research. First, gender inequalities emerged in various aspects of the VCT services and counselors’ experiences. In light of this finding, it is important to rethink the potential of counseling for generating reflections about gender roles, conceptions of safety and prevention in sexual-affective relationships [23]. Studies have found connections between women revealing a positive HIV result to their partners and then suffering physical and sexual violence [40–44]. These same studies have also pointed to a lack of policies and support in VCT services. Future research should explore contexts of violence in testing services more carefully. In addition, cross-sector and interdisciplinary actions beyond the health sector are necessary on a global and local scale to address connections between gender violence and HIV/AIDS [45]. Such actions and interdisciplinary approaches have yet to be consolidated in the VCT services included in this study.

A second area for further research is the place of VCT services in a combination approach to HIV prevention. A combination prevention approach entails integrating efforts among governments, civil society and the scientific community. Social actors who work in STD/AIDS prevention and care networks, such as the VCT services, occupy a privileged place to promote connections between new prevention technologies, psychosocial and community technologies (through processes of listening and social participation) and structural actions (with a focus on improving populations’ sociocultural and economic conditions) [35]. Questions worth exploring include: How might VCT services be reorganized within a vulnerability framework that also contemplates such a combination approach? How could counselors’ practices be reformulated to better address their clients’ sociocultural and economic conditions?

## Limitations

This study’s primary limitation is the reconstruction of counselors’ practices from their narratives. Certainly, observation of in situ counseling or simulated situations would offer deeper indications of their communication and counseling techniques. Requesting those interviewed to recreate their interactions minimized this limitation.

## Conclusions

Efforts to improve counseling as a psycho-social intervention should consider the inadequacy of universal interventions and those that emphasize individual responsibility within the context of the re-medicalization of HIV prevention [35, 46, 47]. VCT services have the potential to promote synergetic individual-community level approaches that strengthen counselors’ capacities for cross-sector responses to HIV. In this sense, VCT services can also contribute to addressing the structural conditions that impede service access to the groups most vulnerable to HIV.

Our empirical strategy established connections between structural factors, the contexts of practices, and agents’ narratives. By including a diverse group of counselors and VCT services in the state of Rio de Janeiro, we reinforced the importance of creating institutional contexts suitable to social needs. Our research points to the importance of further studies about *habitus* formed through health practices and its mutually constitutive relationship with structure [27, 48]. We expect the findings to be useful for future critical analyses of Brazil’s current testing policies within a global context of STD/AIDS prevention policy.

## Abbreviations

ART: Anti-retroviral therapy
CITC: Client-Initiated HIV Testing and Counseling
MSM: Men who have sex with men
TasP: Treatment as Prevention
UNGASS: The United Nations General Assembly Special Session on HIV/AIDS
VCT: Voluntary Counseling and Testing
